# Determinants to Tele-Mental Health Services Utilization Among California Adults: Do Immigration-Related Variables Matter?

**DOI:** 10.1007/s10903-024-01628-z

**Published:** 2024-09-05

**Authors:** Hafifa Siddiq, Kristen R. Choi, Nicholas Jackson, Altaf Saadi, Lillian Gelberg, Ninez A. Ponce, Sae Takada

**Affiliations:** 1 Mervyn M. Dymally College of Nursing, Charles R. Drew University of Medicine and Science, Los Angeles, CA USA; 2 Division of General and Internal Medicine and Health Services Research, Department of Medicine, David Geffen School of Medicine at UCLA, Los Angeles, CA USA; 3 School of Nursing, UCLA, Los Angeles, CA USA; 4 Department of Health Policy and Management, Fielding School of Public Health, UCLA, Los Angeles, CA USA; 5 Department of Neurology, Massachusetts General Hospital, Harvard Medical School, Boston, MA USA; 6 Department of Family Medicine, David Geffen School of Medicine at UCLA, Los Angeles, CA USA; 7 Office of Healthcare Transformation and Innovation, VA Greater Los Angeles Healthcare System, Los Angeles, USA

## Abstract

To investigate the relationship of predisposing, enabling, need, and immigration-related factors to tele-mental health services utilization among California adults, we conducted a secondary analysis of two waves of the California Health Interview Survey (CHIS) collected between 2015 and 2018 (*N* = 78,345). A series of logistic regression models were conducted to examine correlates and predictors to tele-mental health services use. Approximately 1.3% reported the use of tele-mental health services. Overall, health insurance status, severe psychological distress, perceived need for mental health services, and identifying as Asian, remained strong predictors for tele-mental health service use. When accounting for all factors, we found that being a non-citizen was associated with lower odds of tele-mental health service use (AOR = 0.47, CI = 0.26, 0.87, *p* < 0.05). These findings suggest that citizenship, resources to access, and perceived need for mental health care collectively are the most significant factors driving the use of tele-mental health services. There is a need to address inequitable access to tele-mental health services among immigrants who do not qualify for healthcare coverage due to citizenship status.

## Introduction

The rapid expansion of tele-mental health during the COVID-19 pandemic provides an opportunity to address the mental health needs of marginalized populations, including immigrants [1]. Mental health disorders are leading causes of disability and medical comorbidity in the United States (US) [2, 3]. However, immigrants’ utilization remains low due to barriers like difficulty navigating the US healthcare system, understanding mental health care coverage benefits [4], identifying providers, and interacting with them [5–7]. Tele-mental health care delivery (the provision of mental and behavioral health services through technology) may improve access, reduce cost, and increase flexibility, potentially overcoming barriers immigrants face [1]. There is robust evidence that tele-mental health care effectively addresses mental health concerns like depression, substance use disorders, and anxiety across various modalities [8]. However, its utilization among immigrant populations is not well understood.

Socioeconomic and cultural factors have been studied as potential barriers to mental health care among immigrants [7, 9]. Level of education, income, and health insurance may partly explain disparities in mental health care utilization [10]. Understanding the influence of immigration-related factors, such as generation status, language proficiency, and citizenship status may be critical for resource allocation and identification of disparities. Examining these diverse characteristics of US immigrants answers calls for data disaggregation and categorization related to migrant populations [11].

Certain sub-groups of immigrants may have higher unmet mental health care needs [12, 13]. Although immigrants generally report better mental health than US-born counterparts, this varies based on immigration-related factors, like social position and language proficiency [10]. For example, first-generation immigrants who were born outside the US often have differing mental health beliefs and perspectives about treatment [14]. First-generation immigrants also have higher level of unmet needs [4] and may be deterred by cultural insensitivity among mental health care providers [6]. Stigma, discouragement from family or social networks, and restricted socioeconomic opportunities may also affect access [15]. In contrast, second-generation immigrants (US-born with immigrant parents), exhibit higher levels of mental illness or psychological distress [15] and substance-use disorders [16], and utilize mental health services more than first-generation immigrants.

Language proficiency plays a role in mental health care access. Patients with limited English proficiency may face challenges in accessing tele-mental health services due to lack of linguistically appropriate technology, availability of virtual interpreters, and privacy concerns [17]. For example, earlier research found that individuals with limited-English proficiency with mental health disorders were significantly less likely to identify a need for mental health services, experience longer duration of untreated disorders, and use fewer specialty mental health care services [18]. A more recent study found that patients with limited English proficiency had lower rates of overall telehealth use (4.8% versus 12.3%) when compared with proficient English speakers [19]. These studies suggest the importance of focusing on English language proficiency as a critical factor to promote equitable access to telehealth services.

Both foreign-born status and citizenship status are associated with rights, benefits, resources, psychological status as well as health service utilization [20]. The inequality and stress created by immigration law and law enforcement may generate disparities in health among immigrants with different citizenship statuses. Undocumented immigrants are at risk for not receiving needed healthcare services and are disproportionately affected [21]. The rights granted through a documentation status influence health through mechanisms of attaining access to resources, health care, security and relief from immigration enforcement by local and federal law enforcement [22].

Most research among US immigrants point to individual level factors that inhibit individuals from accessing care. Limited research explores how broader immigration law and policies generate disparities in access to tele-health services among immigrants with different citizenship statuses. Our study uses data from the California Health Interview Survey (CHIS) to identify predictors of tele-mental health utilization, considering both immigrant-related factors and individual and contextual factors. To frame this study, we use the Behavioral Model for Vulnerable Populations, which describes contextual and individual-level factors (identified as predisposing, enabling, and need factors) that influence the use of health services in underserved populations [23]. This study aimed to examine predictors (predisposing, enabling, need, and immigration-related factors) including various immigration-related factors to tele-mental health services utilization.

## Methods

### Study Population and Sampling

This study was a secondary data analysis of two waves of the California Health Interview Survey (CHIS) collected in 2015-16 and 2017-18. CHIS is the largest state-level population-based data in the US. CHIS is a cross-sectional survey representative of the state’s overall population, including racial and ethnic minority groups. CHIS reduces coverage bias by conducting the survey not just in English, but in Spanish, Cantonese, Mandarin, Korean, Tagalog, and Vietnamese. This survey methodology ensures representation of the limited English speaking immigrant population in California [24, 25]. The UCLA Institutional Review Board (IRB) approved the analyses of confidential CHIS data (UCLA IRB #11-002227).

### Theoretical Model

The Behavioral Model for Vulnerable Populations is a framework designed to understand health and health-seeking behavior of vulnerable populations [26], and has been applied to work with a variety of populations, including immigrants [27], people experiencing homelessness [26], and people who use drugs [28]. The model consists of three domains. The Predisposing domain consists of demographic characteristics, such as age and sex, and social structure characteristics such as race/ethnicity, educational attainment, and employment. The Enabling domain consists of personal and family resources, such as insurance status and income, and community resources such as region that serve as barriers or facilitators to use of healthcare. The Need domain consists of self-perceptions and objective evaluations of health conditions.

## Measures

### Dependent Variable: Tele-Mental Health Service Use

Tele-mental health services use was determined by asking respondents, “during the past 12 months, did you receive care from a doctor or health professional through a video or telephone conversation rather than an office visit?” If the respondent said yes, they were then asked, “was this care for an emotional or mental health problem?” Respondents who sought tele-mental health were defined as those who answered “yes” to both questions. Respondents who did not seek tele-mental health services were defined as those who did not obtain telehealth care or sought telehealth care for other reasons besides mental health.

### Independent Variables: Predisposing, Enabling, and Need Factors

#### Predisposing Factors

We included the following demographics and social structures: self-reported age (categorized into ages 18–24, 25–34, 35–44, 45–54, 55–64, and 65+), self-reported gender (male or female), educational attainment (high school and below, and some college and above), and employment status (employed and unemployed). Race and ethnicity (non-Hispanic White, Latino/Hispanic, non-Hispanic Black/African American, non-Hispanic American Indian/Alaska Native, non-Hispanic Asian, and all others) were categorized according to the Office of Management and Budget standards to reflect socially constructed identities and the legacy of racism towards people of color [29].

#### Enabling Factors

We examined the following personal/family resources: health insurance status (insured and uninsured), income (family income below the poverty line at 0–99%, 100–199%, 200–299%, and over 300%), and urbanicity, according to the Office of Rural Health Policy (urban and rural status).

#### Need Factors

Psychological distress (severe or not severe) is a dichotomized variable based on a continuous measure of generalized psychological distress during the past year based on the Kessler 6-Item Psychological Distress Scale (K6) [30]. A K6 score of 13 or above was used as the cutoff for severe psychological distress as prior literature has identified a minimum score of 13 as a valid measure indicative of moderate level distress [31]. Perceived need for mental health services is a dichotomized variable based on a question asking respondents “was there ever a time during the past 12 months when you felt that you might need to see a professional because of problems with your mental health, emotions or nerves, or your use of alcohol or drugs?”

#### Immigration-Related Factors

Three different, but inter-related immigration-related variables (citizenship status, generation status, and English-language proficiency) were each examined separately. Citizenship status was determined with a question asking, “in what country were you born?” and “are you a citizen of the United States?” Adults were categorized as non-citizens, naturalized citizens, and US citizens. Non-citizens in this study include both residents and individuals with and without authorization. Generation status was assessed using two measures: foreign-born status (whether they were born outside the US or US-born), mother’s country of birth (foreign-born or US-born), and father’s country of birth (foreign-born or US-born). Adults were assigned as first-generation if they were foreign-born, assigned second-generation if at least one parent was foreign-born, and third-generation if they were US-born with US-born parents. English language proficiency was dichotomized as high (very well and well responses) and low (not well or not at all) English proficiency levels.

### Statistical Analysis

We used summary statistics (e.g. means with SD; relative frequency) to describe the distribution of each independent variable with tele-mental health service use. To assess the strength of the associations, we used bivariate logistic regression models. We then examined the impacts of predisposing, enabling, and need based predictors of tele-mental health, we employed a series of multivariable logistic regression models. Models were specified separately for predisposing, enabling, and need based factors, with a final composite model containing all three factors incorporated simultaneously. A final model incorporated an additional component, immigration-related factors, to the composite model. All analyses were weighted and accounted for the complex survey design of the CHIS. Analyses were conducted using Stata version 17, Stata Corp LLC (College Station, Texas).

## Results

### Descriptive Statistics

Descriptive statistics for the analytic sample are presented in Table 1. In this sample (*N* = 78,345), approximately 51% of respondents identified as female, with a mean age of 55 years. The sample included the following race and ethnicity groups: NH White (42%), Latinx/Hispanic (36%), NH Asian (14%), NH African American/Black (5.6%), NH American Indian/Alaska Native (0.4%), and all other groups (2.5%). Approximately 39% of the population had below high school level education and 37% were unemployed. Regarding generational status, around 32% were foreign-born adults, 20% were US-born adults with immigrant parents, 47% were US-born adults with US-born parents. Regarding citizenship status, 68% were US-born citizens, 17% were naturalized citizens, and 15% were non-citizens. Around 15% of the population reported low English language proficiency. Around 52% of the population lived 300% above the FPL, 9% were uninsured, and 6% lived in rural regions. About 9.7% had severe psychological distress and about 19% perceived the need for mental health care. Approximately 1.3% (*N* = 1,014) reported the use of tele-mental health services.

**Table 1:** Descriptive statistics: predisposing, enabling, need and immigration-related characteristics of California adults on use of tele-mental health service use measured at the bivariate (*N* = 78,345)

Characteristics^a^	Total %
Dependent Variable	
Use of tele-mental health service	1.3
Predisposing Factors	
Age	
18–24	16.0
25–34	20.3
35–44	12.7
45–54	17.5
55–64	16.5
65+	17.0
Gender	
Male	48.9
Female	51.1
Race/ethnicity	
White, non-Hispanic	41.5
Latinx/Hispanic	35.7
Black/African American, NH	5.6
American Indian/Alaskan Native, NH	0.4
Asian only, non-Hispanic, NH	14.3
Native Hawaiian/Other	2.5
Educational attainment	
High school and below	38.8
Some college and above	61.2
Employment status	
Employed	63.5
Not employed	36.5
Generation status	
Foreign-born adult	32.2
U.S.-born with immigrant parent	20.4
U.S. born w/o immigrant parents	47.3
Citizenship status	
US citizen	67.8
Naturalized citizen	17.7
Non-citizen	14.6
Limited English Proficiency (LEP) Status	
Speaks English well/very well	85.2
Speaks English not well/not at all	14.8
Enabling Factors	
Poverty level	
0–99%	16.3
100–199%	18.1
200–299%	13.3
>=300%	52.1
Insurance status	
Uninsured	9.0
Insured	91.0
Urbanicity	
Urban	94.0
Rural	6.0
Need Factors	
Distress level	
Not severe (Kessler < 13)	90.4
Severe (Kessler > = 13)	9.6
Perceived need for mental health services	
No	81.2
Yes	18.7

^a^ Data based on the California Health Interview Survey, 2015–2018

### Characteristics of Tele-Mental Health Users and Non-Users

Bivariate analyses are presented in Table 2. Among predisposing factors, age groups over the age of 45 and identifying as Hispanic/Latinx or Asian were associated with lower odds of tele-mental health services use. Identifying as female and being unemployed were associated with higher odds of tele-mental health services use. Among enabling factors, having health insurance was associated with higher odds of tele-mental health services use. Need factors of having high level of psychological distress and perceived need for mental health services were associated with the use of tele-mental health. Among immigration-related factors, being US-born with or without US-born parents were associated with higher odds of tele-mental health use, while having a lower level of English language proficiency, and being naturalized citizens or non-citizens were associated with lower odds of tele-mental health services use (Table 3).

### Multivariate Analysis

#### Model Predicting Tele-Mental Health Service Use without Immigration-Related Factors

Table 2 presents the multivariate logistic regression results for predisposing, enabling, and need factors (without immigration-related factors) on the reported use of tele-mental health services. Model 1 includes predisposing factors and showed that older-age groups, identifying as Latinx/Hispanic and identifying as Asian were associated with lower odds of use of tele-mental health services. Being unemployed, [adjusted odds ratio (AOR) = 1.36, *p* < 0.05] was associated with higher odds of use of tele-mental health services.

**Table 2:** Logistic regression of predisposing, enabling, and need factors on California adults’ use of tele-mental health services (*N* = 78,345)

Characteristics	Bivariate associations:				Model 1:		Model 2:		Model 3:		Model 4: Predisposing + enabling + need factors and use of tele-mental health services	
					Predisposing factors and use of tele-mental health services		Enabling factors and use of tele-mental health services		Need factors and use of tele-mental health services			
	**OR**		**95% CI**		**AOR**	**95% CI**	**AOR**	**95% CI**	**AOR**	**95% CI**	**AOR**	**95% CI**
Predisposing Factors												
Age												
18–24	Ref				Ref						Ref	
25–34	0.87		[0.59, 1.27]		0.86	[0.58, 1.27]					0.93	[0.63, 1.39]
35–44	0.82		[0.51, 1.23]		0.81	[0.53, 1.25]					1.11	[0.72, 1.71]
45–54	0.59		[0.38, 0.92]*		0.56	[0.35, 0.90]**					0.89	[0.55, 1.43]
55–64	0.51		[0.35, 0.74]**		0.41	[0.28, 0.60]***					0.74	[0.50, 1.11]
65+	0.49		[0.32, 0.76]***		0.3	[0.18, 0.49]***					0.93	[0.57, 1.51]
Gender												
Male	Ref		Ref		Ref						Ref	
Female	1.34		[1.05, 1.69]*		1.27	[0.99, 1.64]					1.02	[0.80, 1.31]
Race/ethnicity												
White, Non-Hispanic	Ref				Ref						Ref	
Latinx/Hispanic	0.69				0.61	[0.43, 0.85]**					0.77	[0.56, 1.07]
Black/African American, NH	0.97				0.9	[0.58, 1.41]					1.08	[0.69, 1.70]
American Indian/ Alaskan Native, NH	0.83				0.77	[0.35, 1.69]					0.69	[0.30, 1.58]
Asian, NH	0.46				0.39	[0.24, 0.63]***					0.59	[0.35, 0.99]*
Other	1.53				1.24	[0.63, 2.44]					1.04	[0.55, 1.9]
Educational												
attainment												
High school and below	Ref				Ref						Ref	
Some college and above	1.29				1.27	[0.94, 1.71]					1.17	[0.86, 1.57]
Employment status												
Employed	Ref				Ref						Ref	
Unemployed	1.35				1.8	[1.36, 2.39]***					1.37	[1.05, 1.78]**
Enabling Factors												
Poverty level												
0–99%	Ref			Ref			Ref				Ref	
100–199%	0.95			[0.65, 1.40]			0.94	[0.64, 1.39]			1.08	[0.73, 1.60]
200–299%	1.07			[0.70, 1.64]			1.05	[0.68, 1.60]			1.19	[0.76, 1.86]
>=300%	0.74			[0.53, 1.02]			0.69	[0.50, 0.96]**			0.86	[0.61, 1.22]
Insurance status												
Uninsured	Ref			Ref			Ref				Ref	
Insured	2.75			[1.63, 4.66]***			2.98	[1.75, 5.09]***			2.36	[1.36, 4.10]**
Urbanicity												
Urban	Ref						Ref				Ref	
Rural	1.25						1.2	[0.86, 1.68]			1.16	[0.81, 1.65]
Need Factors												
Distress level												
Not severe	Ref								Ref		Ref	
(Kessler < 13)												
Severe	8.42								2.53	[1.97, 3.26]***	2.47	[1.91, 3.20]***
(Kessler > = 13)												
Perceived need for mental health services												
No	Ref								Ref		Ref	
Yes	13.35								9.03	[6.69, 12.170***	8.54	[6.45, 11.32]***

*p < 0.05

**p < 0.01

***p < 0.001

**Table 3:** Logistic regression with immigration-related factors on California adults’ use of tele-mental health services (*N* = 78,345)

Immigrant-Related Variables	Bivariate associations		Model 5a: Immigration-related factors controlling for predisposing factors + use of tele-mental health services		Model 5b: Immigration-related factor controlling for predisposing + enabling + need factors	
	**OR**	**95% CI**	**AOR**	**95% CI**	**AOR**	**95% CI**
** Generation status**						
1st (Foreign-born)	Ref		Ref		Ref	
2nd (US-born with immigrant parents)	2.22	[1.45, 3.40]***	1.81	[1.13, 2.91]*	1.21	[0.75, 1.94]
3rd (US-born w/o immigrant parents)	2.54	[1.81, 3.57]***	2.07	[1.28, 3.35]**	1.27	[0.81, 1.97]
** English Language Proficiency (ELP)**						
Higher ELP	Ref		Ref		Ref	
Lower ELP	0.32	[0.16, 0.65]***	0.39	[0.18, 0.85]*	0.66	[0.29, 1.50]
** Citizenship status**						
Citizen	Ref		Ref		Ref	
Naturalized citizen	0.54	[0.36, 0.82]***	0.76	[0.40, 1.41]	1.04	[0.65, 1.65]
Non-citizen	0.23	[0.14, 0.40]***	0.28	[0.11, 0.74]**	0.48*	[0.26, 0.89]

*p < 0.05

**p < 0.01

***p < 0.001

Model 2 includes enabling factors and showed that 300% poverty level (AOR = 0.69, *p* < 0.01) and being insured (AOR = 2.98, *p* < 0.01) were associated with higher odds of use of tele-mental health services. Model 3 includes perceived need and level of psychological distress and showed that having a perceived need for mental health services (AOR = 9.03) and having a severe level of psychological distress (AOR = 2.53, *p* < 0.001), were significantly associated with the use of tele-mental health services. Model 4 includes all variables without considering immigrant-related factors. This final model showed that identifying as Asian, (AOR = 0.59, *p* < 0.05), having a perceived need for mental health services (AOR = 8.54), and having a severe level of psychological distress (AOR = 2.44, *p* < 0.001), were significantly associated with the use of tele-mental health services.

#### Model Predicting Tele-Mental Health Service Use with Immigration-Related Factors

Our Model 5a and 5b added immigration-related factors of generation status, English-language proficiency, or citizenship status with the use of tele-mental health services, adjusting for predisposing, enabling, and need variables. Each immigration-related factor was examined separately (See Table 3). We found that among the three immigrant-related factors, being a non-citizen was associated with lower odds of tele-mental health service use (AOR = 0.48, *p* < 0.05). When considering only predisposing factors, being US-born with one or more immigrant parents (second-generation) or being US-born with both parents born in the US (third-generation) was significantly associated with higher odds of using tele-mental health services (AOR = 1.81, p = < 0.05) and (AOR = 2.07, p = < 0.01) but was no longer significant when enabling and need factors are included in the model. Supplementary Table 4 show our sensitivity analyses examining a model with citizenship status accounting for predisposing, enabling, and need factors without insurance status. We found that non-citizen status remained significantly associated with tele-mental health service use, but naturalized citizens no longer remained significant.

## Discussion

This study examined predisposing, enabling, and need factors contributing to the use of tele-mental health services, focusing on immigration-related factors (i.e. generation status, English-language proficiency, or citizenship status). This study confirmed prior findings that use of tele-mental health services were minimally utilized prior to the COVID-19 pandemic [32, 33]. Non-citizens and naturalized citizens were found to have significantly lower odds of tele-mental health service use when accounting for various factors. The most significant predictors for tele-mental health service use were enabling (insured) and need factors (perceived need for mental health services and severe psychological distress). These findings suggest that resources to access and perceived need for mental health care collectively are the biggest drivers to the use of tele-mental health services than non-modifiable predisposing factors.

### Immigration-Related Factors

Citizenship remained a strong predictor for the use of tele-mental health services, consistent with evidence of lower rate of healthcare utilization among non-citizens in general (including among refugees, asylum seekers, and undocumented immigrants) [21]. It is suggested that societal disadvantages related to non-citizenship in the US form barriers to tele-mental health, emphasizing the need for interventions targeting non-citizens who have unmet mental health care needs. Naturalization is a key indicator of social and political inclusion for immigrants and is related to health [34].

Tele-health provides opportunities for patient access and engagement that could mitigate limitations often experienced by immigrant families. Overall, citizenship status may indicate the overall advantages and privileges of immigrants that have obtained the ability to fully integrate within the US. Citizenship is linked to a person’s strong connection to rights, responsibilities, roles, resources, and relationships and a sense of belonging that is validated by others [35] and can be a form of social capital that promotes integration into the US economy [36], promotes access to healthcare [37], and is overall, a social determinant of health [38].

### Healthcare Coverage for Tele-Mental Health Care

Despite the rapid expansion of tele-mental health services in the US during the COVID-19 pandemic, there remains significant inequity in access to these services. A recent study showed that tele-mental health availability increased from around 12–70% from 2010 to 2020, demonstrating a rapid expansion of services with implications on the need to address equitable access to such services. Historically marginalized groups, including immigrants and refugees, racial and ethnic minorities, and individuals with disabilities, are particularly disadvantaged [39, 40]. Non-citizens live at the intersection of many social, economic and cultural challenges that create a digital divide, defined as the technology, internet, design usability, and empowerment access gaps between marginalized populations and others [41]. Policy measures intended to expand telehealth that overlook the specific needs of immigrants ineligible for federally funded healthcare, may further exacerbate the digital divide marked by disparities in technology access and digital empowerment [42].

### Future Research

With an increasingly diverse immigrant population in the U.S. and the recognition that socio-cultural and political context plays a significant role in shaping mental health experiences and outcomes, there is a need for future research in effectively delivering culturally appropriate tele-mental health care for immigrant populations. Population-based studies have reported different prevalences of depression by race and ethnicity [43–45]. The influence of race and ethnicity on mental health has long been considered in the U.S., as different racial and ethnic groups may have unique cultural beliefs, practices, and values that influence their understanding of mental health, coping mechanisms, and help-seeking behaviors [46] as well as social and structural factors that predispose them to mental illness [15]. Other research highlights the need to increase access to culturally appropriate mental health care in community settings and promoting more social-based therapies especially for immigrant older adults due to cultural beliefs and stigma [47], yet there is a need for further tele-mental health research in virtually meeting the needs of a population with significant mental health needs and limited mental healthcare access.

### Policy Implications

Broadly, federal and state level policies that have been passed amid the COVID-19 pandemic promoted the rapid expansion of telehealth services [48]. Policy recommendations to improve telehealth equity include increasing access to digital technology, digital literacy training and broadband internet [49]. Legislation passed amid the COVID-19 pandemic promoted telehealth services expansion, but may not have adequately promoted tele-mental health services access for immigrants including H.R. 6074: Coronavirus Preparedness and Response Supplemental Appropriations Act [50], H.R. 748: Coronavirus Aid, Relief, and Economic Security (CARES) Act [51], H.R. 133: Consolidated Appropriations Act, 2021 [52], and the H.R. 1319: American Rescue Plan Act of 2021 [53]. While special considerations were made to include funding for rural and under-resourced communities, and building broadband and technological infrastructures, the issues of inequitable access to tele-mental health services among immigrants who do not qualify for healthcare coverage due to citizenship status remains unaddressed. For example, the CARES Act focused on unauthorized immigrants’ access to selected programs [54] without resolving their ineligibility for most federal benefits for social, food, and housing programs [54]. A critical need exists for the expansion of Medicare for new immigrants, and for expanding the definition of authorized immigrants to include DACA recipients. These policy changes may address access issues for immigrants who are systematically disqualified for federally-funded healthcare [19].

There are strengths and limitations to this study. This is the beginning of follow-up studies using CHIS 2019-20, and 2021-22. Strengths include using a large, diverse population-based sample of adults in California to examine tele-mental health use and had measures of multiple immigration-related factors. Before data analysis began, we identified several immigration-related variables available in the CHIS dataset. We constructed models according to an established framework for healthcare utilization to examine the role of predisposing, enabling, and need factors. Limitations of the study include a lack of detailed measures on cultural beliefs of mental health, types of tele-mental health used, or experiences with tele-mental health and that all measures were based on self-reports. It is also difficult to assess cause and effect relationships in a cross-sectional study. Our study used data from 2015 to 2018, before the COVID-19 pandemic when there was a dramatic increase in tele-mental health in the US [55]. As such, there is a need for replication studies to verify whether immigration-related disparities still exist even after expansions of tele-mental health during the pandemic. Findings from California might not be generalizable to other parts of the US, but California is home to the highest proportion (27.8%) and largest population of immigrants (10.5 million) [56].

## Conclusion

Tele-mental health services can potentially address access disparities among US immigrants, but citizenship status is a significant factor influencing utilization of these services. This study finds that prior to the COVID-19 pandemic, non-citizens were less likely to use tele-mental health services than US-born citizens. It emphasizes the need for future research to consider the role of immigration-related factors, especially as overlooking these can exacerbate health inequities among immigrants due to policies and legal barriers.
